# Comparison Between Chemiluminescent Assay and Enzyme-Linked ImmunoSorbent Assay Techniques for the Detection of Anti-Cardiolipin and Anti-β2 Glycoprotein I Antibody Values

**DOI:** 10.3390/diagnostics16111620

**Published:** 2026-05-25

**Authors:** Fulvio Castelgrande, Sergio Bernardini, Marzia Nuccetelli

**Affiliations:** Department of Experimental Medicine, Tor Vergata University, 00133 Rome, Italy; bernards@uniroma2.it (S.B.); marzia.nuccetelli@ptvonline.it (M.N.)

**Keywords:** anti-phospholipid antibodies, antiphospholipid syndrome, anti-cardiolipin antibodies, anti-beta2-glycoprotein I antibodies, enzyme-linked immunosorbent assay, chemiluminescent assay, methodology comparison

## Abstract

**Background**: Antiphospholipid antibodies (aPLs) are essential for antiphospholipid syndrome (APS) diagnosis, which is based on clinical and laboratory parameters, including the detection of lupus-anticoagulant (LAC), anti-cardiolipin (aCL) and anti-β2-glycoprotein-I (aβ2-GPI) antibodies. The enzyme-linked immunosorbent assay (ELISA) is the reference methodology for classification criteria, although chemiluminescence immunoassays (CLIA) are more common in clinical practice. **Methods**: Since LAC reflects the functional activity of a large subset of antiphospholipids, through coagulation assays that enhance a phospholipid-dependent inhibitory effect, it has been used as a reference for assessing ELISA and CLIA reliability. Samples, separated into positive and negative LAC, were selected by CLIA detection in negative and positive IgG/IgM aCL and aβ2-GPI (*cut-off* > 20 U/mL). **Results**: The ELISA/CLIA comparison showed a 100% concordance in triple negative groups, highlighting an optimal analytical specificity; a higher concordance in the aβ2-GPI IgM-positive groups compared to positive aCL IgM (100% vs. 76% in LAC-positive groups; 82% vs. 71% in LAC-negative groups), as well as in aβ2-GPI IgM-negative groups compared to negative aCL IgM in LAC-positive groups (100% vs. 87.5%); and a massive concordance reduction in positive IgG aβ2-GPI and aCL groups (44% vs. 50% in LAC-positive groups; 4.8% vs. 4.5% in LAC-negative groups). Concordance increased in all groups with a higher CLIA cut-off (>50 U/mL). **Conclusions**: Although CLIA performances partly differed from ELISA, this does not preclude their use in APS diagnosis, which aims for higher sensitivity in detecting cases of disease. ELISA is confirmed to be more reliable for classification criteria that aim for high specificity to reduce false positives.

## 1. Introduction

Antiphospholipid antibodies (aPLs) are directed against negatively charged phospholipids or phospholipid-binding proteins and are involved in the diagnosis of antiphospholipid syndrome (APS) [1].

Antiphospholipid syndrome is a systemic autoimmune disease characterized by clinical manifestations, including vascular thrombosis and/or pregnancy morbidity (such as recurrent early miscarriage, fetal loss or preterm birth), and by laboratory tests detecting the persistent presence of antiphospholipid antibodies [2]. It is well known that aPLs are not specific to APS as they can also be found in healthy individuals or induced by bacterial or viral infections [3]. The mean age at APS diagnosis is around 50–55 years, with a slight female predominance, an estimated incidence of 1–2 cases per 100,000 person/years, and a prevalence of 40–50 cases per 100,000 [4,5,6].

The diagnosis is based on Sapporo criteria, first published in 1999 [7] and revised in Sidney in 2006 [8]. These criteria consist of clinical and laboratory domains. The clinical domain includes a vascular domain (showing venous and/or arterial thrombosis that can affect any organ) and an obstetric domain (showing unexplained fetal deaths, preterm births, and one or more consecutive spontaneous abortions); the laboratory domain includes the detection of lupus anticoagulant (LAC), IgG and/or IgM anti-cardiolipin antibodies (aCL) and anti-β2 glycoprotein I antibodies (aβ2-GPI). The diagnosis of APS is confirmed when at least one clinical criterion and one laboratory criterion are met [8,9,10].

Lupus anticoagulant, first discovered in systemic lupus erythematosus (SLE) patients, is detected by coagulation-based assays and represents the aPLs parameter most strongly associated with thrombotic events; it reflects the ability of aPLs to interfere with phospholipid-dependent coagulation reactions, and particularly with the conversion of prothrombin to thrombin, thereby preventing clot formation in vitro [11]. LAC determination follows the guidelines established by the International Society on Thrombosis and Haemostasis (ISTH) [12].

Cardiolipin (CL) is a negatively charged phospholipid primarily located in the inner mitochondrial membrane and exposed following cell injury or apoptosis [13]. The sensitivity of aCL testing for APS is relatively high, whereas its specificity is generally lower, as these antibodies can also be detected in other autoimmune diseases and infections [14,15]. An important aspect to consider is that aCL antibodies recognize cardiolipin in a β2-GPI-dependent manner, as β2-GPI binding to anionic phospholipids such as CL can expose the antigenic epitopes recognized by these auto-antibodies [16].

On the other hand, β2-GPI is an apolipoprotein of approximately 50 kDa, where glycosylations are included, produced by endothelial cells, hepatocytes, and trophoblast cells, organized into five domains (D1–D5) [17,18]. It can assume multiple conformations (“closed”, “open” and “intermediate”), and interaction with anionic surfaces can expose the immunodominant epitope on D1. Anti-β2-glycoprotein I antibodies are characterized by lower sensitivity but significantly higher specificity for APS, compared to aCL [17,18].

In 2023, the American College of Rheumatology (ACR), in collaboration with the European League Against Rheumatism (EULAR), published new APS classification criteria to use in research settings such as observational studies and trials [10]. Unlike the previous Sapporo criteria, these include an entry criterion requiring at least one positive test for antiphospholipid antibodies within three years of a clinical event, followed by weighted additive criteria grouped into six clinical domains and two laboratory domains. Patients accumulating at least three points in each of the clinical and laboratory domains are classified as having APS [10]. These new criteria introduced semiquantitative *cut-offs* based on an enzyme-linked immunosorbent assay (ELISA) for aCL IgG/IgM and anti-β2-GPI IgG/IgM, expressed in phospholipid units (GPL/MPL) and indicated as “moderately high” (40–79 units) and “high” (>80 units). Importantly, the criteria allow for the use of alternative analytical platforms, such as chemiluminescence immunoassay (CLIA), provided that equivalent *cut-offs* are defined according to the ISTH standardization procedures [10]. However, few studies have directly compared CLIA and ELISA, so clear *cut-off* values and the corresponding ranges between platforms have not been defined [19,20].

Given that for routine clinical practice, most major pathology and clinical biochemistry laboratories use faster and fully automated chemiluminescence platforms, we compared CLIA and ELISA techniques to test IgG, IgM aCL, and aβ2-GPI levels.

Laboratory tests for lupus anticoagulant reflect the functional activity of a large subset of antiphospholipid antibodies; these tests are based on the inhibitory activity of plasma on coagulation in phospholipid-dependent assays, which is due to the presence of antiphospholipid antibodies that prolong the phospholipid-dependent clotting time. For this reason, we decided to exploit the LAC test in the experimental design as a reference for assessing ELISA and CLIA reliability. These samples were separated into two principal categories: positive and negative LAC. Each of them was divided into eight subgroups (aCL IgG/IgM-negative and -positive and aβ2-GPI IgG/IgM-negative and -positive), determined via CLIA detection, using the manufacturer’s *cut-off* value of 20 U/mL. Our study aims to verify the reliability of the different methodologies and to determine whether a correlation exists that can be used to assess the positivity ranges.

## 2. Materials and Methods

### 2.1. Patients and Samples

This study was performed at the clinical biochemistry laboratory of “Tor Vergata” University Hospital in Rome, in accordance with the Declaration of Helsinki and with local ethical guidelines.

Serum samples undergoing routine laboratory detection of aPLs, as requested by physicians, were collected from March 2024 to March 2025 and selected based on the levels of IgG/IgM aCL and IgG/IgM aβ2-GPI, obtained using the BioFlash automated chemiluminescence analyzer (Werfen, Inova Diagnostics, San Diego, CA, USA), and from LAC values measured using the CoagOne automated coagulometer (Stago, Asnieres sur Seine, Paris, France). All samples were tested for LAC, IgG/IgM aCL and IgG/IgM aβ2-GPI. Subsequently, samples were categorized as LAC-positive or LAC-negative. Each LAC group was further divided into four subgroups based on the negativity of IgG, IgM aCL, and aβ2-GPI, and on the isolated CLIA positivity of IgG, IgM aCL, and aβ2-GPI, using the manufacturer’s *cut-off* value of 20 U/mL. The number of samples, mean age and the total male/female number, as well as the male/female number for each subgroup, are detailed in Table 1.

### 2.2. Lupus Anticoagulant Assay

Lupus anticoagulant (LAC) detection was performed on the fully automated CoagOne coagulometer (Stago, Asnieres sur Seine, Paris, France).

Plasma samples were stored at −20 °C and thawed 30 min before processing. LAC testing was performed within a week from storage.

Laboratory tests for lupus anticoagulant are based on coagulation assays that enhance a phospholipid-dependent inhibitory effect.

The phospholipid-dependent tests used are the dRVV (diluted Russell’s viper venom test) and the aPTT-LA (activated partial thromboplastin time for lupus anticoagulant), which include a *screening* test and a confirmatory test. The results are expressed as a ratio between plasma under examination and a pool of normal human plasma supplied by the company. The presence of LAC is strongly suspected when both tests yield a *screen* ratio greater than 1.20. To determine if the value is caused by the presence of antiphospholipid antibodies, a confirmatory test is performed using a lyophilized preparation of phospholipids. If, in the tested plasma, there is the presence of antiphospholipid antibodies, they will bind to the phospholipids mixture and there will be a correction of the prolonged clotting time, giving a ratio <1.20.

The final result is then expressed as the normalized ratio:

$$\mathrm{Normalized}\;\mathrm{ratio}\;=\;\frac{Screen\;ratio}{Confirm\;ratio}$$

LAC is considered positive when the normalized ratio in both tests is ≥1.20.

### 2.3. Detection of Anti-Cardiolipin and Anti-β2-Glycoprotein I IgG and IgM Antibodies by Chemiluminescence Assay

For the anti-cardiolipin and anti-β2-glycoprotein I determinations, the QUANTA Flash^®^ aCL IgG/IgM and β2-GPI IgG/IgM kits (Inova Diagnostics, San Diego, CA, USA) were used. The assays were performed on the fully automated BIO-FLASH instrument (Inova Diagnostics, San Diego, CA, USA).

Chemiluminescence assay is based on magnetic beads coated with purified bovine cardiolipin/human β2-glycoprotein I complex for the aCL detection and purified human β2-glycoprotein I for the aβ2-GPI detection. In the first step, 30 µL of samples (diluted 1:10) are incubated at 5 °C for 9.5 min; after magnetic separation and washing, a tracer consisting of isoluminol-labeled antibodies is added to bind the patient’s antibodies captured by the beads, and incubated for 9.5 min. Finally, the luminescent reaction is activated; the emitting light is measured at 440 nm by the optical system and reported as relative light units (RLUs). The system uses a lot-specific working calibration curve, often defined by a 4-parameter logistic calibration curve, to translate RLUs into analyte concentrations. Only one calibration is required per lot of reagents, as the machine maintains a stable working curve for the life of the lot. The RLUs are directly proportional to the specific antibodies present in the sample. The manufacturer’s positive value is >20 U/mL.

### 2.4. Detection of Anti-Cardiolipin and Anti-β2-Glycoprotein I IgG and IgM Antibodies by Enzyme-Linked Immunosorbent Assay

Enzyme-linked immunosorbent assay determinations of aCL and aβ2-GPI were performed manually using QUANTA ${Lite}^{®}$ ACA IgG III, ACA IgM III, β2-GPI IgG and β2-GPI IgM kits (Inova Diagnostics, San Diego, CA, USA).

Serum samples were stored at −80 °C and thawed one hour before processing.

Briefly, samples were diluted 1:101 by adding 5 µL in 500 µL of sample diluent (tris-buffered saline, tween 20, protein stabilizers and preservatives) according to the manufacturer’s instructions. One hundred microliters of calibrators (ranging from 0 to 150 GPL/MPL), diluted sera, negative and positive controls, were incubated (30 min, room temperature) on a microtiter plate adsorbed with the specific antigen. After 3 washing steps with 300 µL tris-buffered saline and tween 20 solution, 100 µL of horse radish peroxidase conjugate was added to each well and incubated for 30 min at room temperature. Subsequently, 3 washing steps were performed and 100 µL of tetramethylbenzidine chromogen was added for 30 min incubation at room temperature to develop a colorimetric reaction. Finally, through the addition of 100 µL of a stop solution (0.344 M sulfuric acid), the absorbance was read at 450 nm.

The manufacturer’s reference values are: 20–79 GPL/MPL, moderate positive; ≥80 GPL/MPL, strong positive.

### 2.5. Main Analytical and Diagnostic Characteristics of Enzyme-Linked Immunosorbent Assays and Chemiluminescence Assays

A comparison of the ELISA and CLIA characteristics, as declared by the manufacturer, is reported in Supplementary Table S1, specifically: antigen source, diagnostic specificity, diagnostic sensitivity, coefficient of variation (inter-assay and intra-assay) and range of detection. The antigen source is the same in both CLIA and ELISA for aβ2-GPI IgG and IgM (purified β2-GPI protein from human serum); in aCL IgG and IgM, the cofactor is different between ELISA (purified bovine cardiolipin as an antigen and bovine β2-GPI protein as cofactor) and CLIA (purified bovine cardiolipin as an antigen and human β2-GPI protein as cofactor). Diagnostic specificity is higher than 90% for all the detected antibodies (the best values are obtained with the ELISA kits, where the lowest value is 93.6%). Diagnostic sensitivity shows great variability. The best values are reported for aCL IgG and IgM detected by ELISA kits (96.6% vs. 94%, respectively); aβ2-GPI detected by ELISA shows an 83.3% value for the IgM class and a lower value (20.8%) for the IgG class. Regarding the diagnostic sensitivity of the CLIA kits, the values decrease compared to ELISA, except for aβ2-GPI IgG (54.3% vs. 33.7% for aCL IgG and IgM, respectively; and 64.1% vs. 29.3% for aβ2-GPI IgG and IgM, respectively). The coefficient of variation (inter-assay and intra-assay) for all the kits is less than 10%, with the exception of the aCL IgM ELISA kit (inter-assay: 12.0%; intra-assay: 12.2%). Usually, values lower than 20% represent good data consistency and indicate low analytical variability. In the end, due to the high analytical sensitivity of CLIA, the range of detection is much higher than that of ELISA: 2.6–2024 U/mL, 1–774 U/mL, 6.4–6100 U/mL, and 1.1–841 U/mL for each of aCL IgG and IgM and aβ2-GPI IgG and IgM, respectively, compared to 0–150 U/mL for all the ELISA kits. Note that analytical sensitivity refers to the ability of a test to detect analytes at low concentrations; therefore, even with low sample volumes, it can give very high signals. Diagnostic sensitivity is the ability of a test to discriminate individuals with a disease from those without it.

### 2.6. Statistical Analysis

Data were analyzed with GraphPad Prism Software (Boston, MA, USA; version: 8.0.1.244).

Since the samples have been selected based on positive or negative CLIA results, the classical 2 × 2 contingency table to calculate the Coehn’s kappa value, could not be used.

Therefore, the concordance value of each group (reported as a percentage) was calculated using the matrix reported in Table 2:

$$\mathrm{Concordance}=\frac{a+d}{a+b+c+d}$$

Quantitative correlation to assess the rho (ρ) coefficient of agreement between ELISA and CLIA results was performed using Spearman rank correlation for non-parametric data. A *p*-value < 0.05 was considered statistically significant.

A post hoc power analysis using McNemar’s test was performed to determine differences between ELISA and CLIA results. The analysis was based on the observed discordant values and was conducted using G*Power, version 3.1.9.7 (Heinrich Heine University, Düsseldorf, Germany). An alpha error probability of 0.05 was adopted and a statistical power of (1 − β) ≥ 0.80 was considered adequate to evaluate the reliability of the study results. The analysis was performed on the total number of samples (349), due to CLIA-based selection of the groups.

## 3. Results

A total of 349 samples were analyzed, showing, as expected, a higher number of women compared to men (247 vs. 102; 2.42:1 F/M ratio). The mean age was 50.6 years ± 14.68 years.

Samples were separated into two main categories (LAC-positive and -negative) and further selected by CLIA-negative or -positive results (*cut-off* > 20 U/mL) (Table 1).

Each subgroup has been detected by ELISA, and CLIA/ELISA concordance values were calculated.

**Figure 1:**
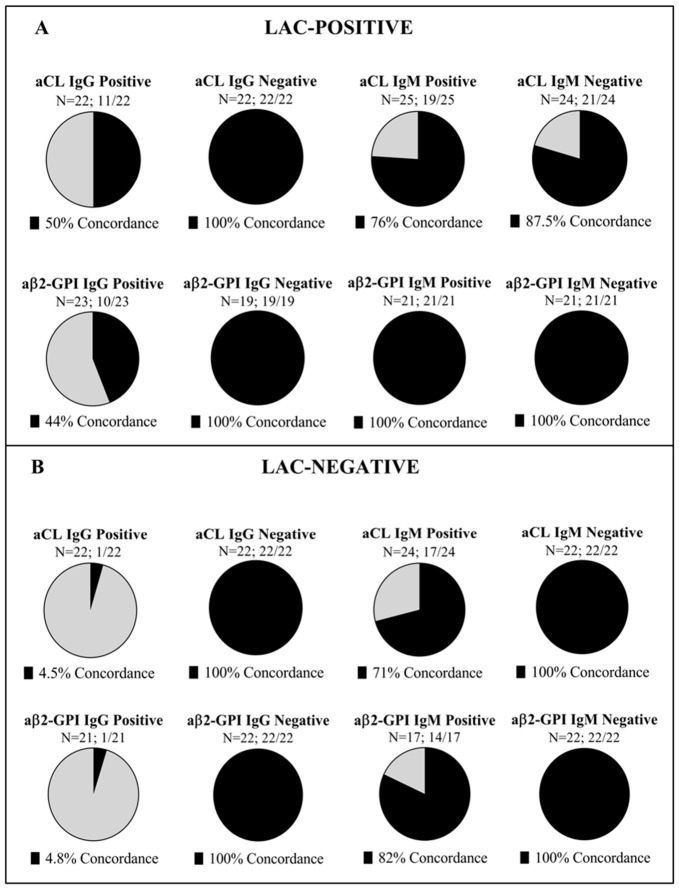
Concordance in the CLIA/ELISA comparison, expressed as percentage values. The percentage values of each group are represented in the form of pie charts. Concordance is depicted in black. Panel (**A**): anti-β2-glycoprotein I IgG/IgM and anti-cardiolipin IgG/IgM concordance in positive LAC groups. Panel (**B**): anti-β2-glycoprotein I IgG/IgM and anti-cardiolipin IgG/IgM concordance in negative LAC groups.

In the LAC-positive group, anti-cardiolipin IgG showed a concordance of 50% in CLIA-positive samples (11/22), whereas a 100% concordance was observed in CLIA-negative samples (22/22); anti-cardiolipin IgM showed a concordance of 76% in CLIA-positive samples (19/25) and 87.5% in CLIA-negative samples (21/24); anti-β2-glycoprotein I IgG antibodies showed a concordance of 44% in CLIA-positive samples (10/23) and a 100% concordance in CLIA-negative samples (19/19); and anti-β2-glycoprotein I IgM antibodies displayed a 100% concordance in both CLIA-positive and CLIA-negative samples (21/21 for both) (Figure 1, panel A).

In the LAC-negative group, a markedly lower concordance was observed for anti-cardiolipin IgG in CLIA-positive samples (4.5%; 1/22), whereas a 100% concordance was observed in CLIA-negative samples (22/22); anti-cardiolipin IgM antibodies showed a concordance of 71% in CLIA-positive samples (17/24) and 100% in CLIA-negative samples (22/22); anti-β2-glycoprotein I IgG also showed a very low concordance in CLIA-positive samples (4.8%; 1/21), while maintaining a 100% concordance in CLIA-negative samples (22/22); and anti-β2-glycoprotein I IgM demonstrated a concordance of 82% in CLIA-positive samples (14/17) and 100% in CLIA-negative samples (22/22) (Figure 1, panel B).

Since CLIA assays generally show a higher analytical sensitivity [21,22], the results were re-evaluated by maintaining the same ELISA *cut-off* (>20 U/mL) and considering a *cut-off* > 50 U/mL for the aPLs positive groups. The concordance values increased in all groups; the lowest values remained in LAC-negative IgG groups. In particular, the aCL IgG group went from 4.5% to 14% and from 50% to 65% in negative and positive LAC, respectively; the aCL IgM group went from 71% to 83% and from 76% to 89% in negative and positive LAC, respectively; the aβ2-GPI IgG group went from 4.8% to 15% and from 44% to 59% in negative and positive LAC, respectively; and the aβ2-GPI IgM group went from 82% to 89% in negative LAC and remained 100% in positive LAC (Table 3).

Data were also analyzed by Spearman regression to assess the correlation between CLIA and ELISA methods. Good and strong correlations (rho > 0.6) were found in some LAC-positive groups with statistically significant *p*-values. Surprisingly, LAC-positive groups with a 100% concordance showed a lower correlation, with non-statistically significant *p*-values. In LAC-negative groups, rho values were all <0.6, showing a moderate correlation in aCL IgM-positive groups, and a weak and very weak correlation in aCL Ig-positive groups. The *p*-values followed the same LAC-positive groups trend: they were statistically significant with a moderate correlation and non-statistically significant with a weak correlation (Supplementary Table S2). The post hoc power analysis showed a statistical power value (1 − β) = 1 that excludes a random association between ELISA and CLIA results.

## 4. Discussion and Conclusions

Laboratory data on antiphospholipid syndrome are essential to guide clinicians towards a correct diagnosis and risk assessment. Over the years, several efforts have been made to refine the classification. The ISTH proposed more stringent and detailed criteria, emphasizing thrombotic risk and the importance of auto-antibody levels, introducing the “triple positive” result (the simultaneous presence of LAC, aCL and aβ2-GPI) as a very high thrombosis risk factor [23]. In addition, due to high inter-assay variability, they recommended that each laboratory should evaluate laboratory-specific *cut-offs* based on the non-parametric 99th percentile of reference individuals. Subsequently, ACR/EULAR developed new classification criteria, which are based on clinical and laboratory domains [10]. Despite the different revised guidelines, there is agreement on the reference methodology for aPLs detection, which should be the ELISA test because, so far, the levels corresponding to “moderate” and “high” have not been standardized with chemiluminescence platforms.

Our CLIA/ELISA comparison data showed an optimal analytical specificity in negative LAC groups, with a 100% concordance, both in the aCL- and in the aβ2-GPI IgG- and IgM-negative samples. Those data are important in underlining the finding that both techniques are able to exclude the possibility of detecting false positives, confirming patients triple negativity and reflecting the high diagnostic specificity values declared by the company (>90.8%).

Moreover, even if the assays for the aβ2-GPI and aCL IgM class are reported to have similar diagnostic sensitivity and specificity values, a general trend of a higher concordance in aβ2-GPI IgM groups has been observed, compared to aCL IgM: 82% vs. 71% in positive groups, with negative LAC; 100% vs. 87.5% and 76% for aCL-negative and -positive groups, respectively, with positive LAC (Figure 1). These findings are consistent with several previous studies, reporting that anti-β2-glycoproteins are more accurate in APS diagnosis than anti-cardiolipins, commonly detectable in other conditions [14,24,25].

Nevertheless, although there was a 100% concordance in IgG aβ2-GPI- and aCL negative-groups with negative and positive LAC, the most striking finding concerns the IgG detection in aβ2-GPI- and aCL-positive groups, where a massive reduction in the concordance rate has been observed. In fact, LAC-positive groups showed a 44% concordance in aβ2-GPI samples and a 50% concordance in aCL samples; in LAC-negative groups, the concordance is drastically reduced to 4.8% in aβ2-GPI samples and 4.5% in aCL samples. Evaluating the diagnostic sensitivity values reported by the company, a large discrepancy emerges that could explain these low concordances (aCL IgG: ELISA 96.6%, CLIA 54.3%; aβ2-GPI IgG: ELISA 20.8%, CLIA 64.1%).

Note that a general trend of a higher IgM concordance with respect to the IgG class has been observed in almost all groups, as also described in some comparative studies, reporting CLIA and ELISA to be in close but incomplete concordance for the detection of aCL and aβ2-GPI antibodies [19,20]. Indeed, a higher concordance was observed for aCL IgG/IgM and aβ2-GPI IgM antibodies, and significantly lower concordance was observed for aβ2-GPI IgG antibodies. A possible explanation could be linked to the polyreactivity of the IgM class, which, in its pentameric form, can isomerize and assume different conformations [26].

Good Spearman correlations were found in the majority of LAC-positive groups, but surprisingly not in the groups with 100% concordance. This makes it evident, as expected, that it is difficult to compare different methodologies, with distinct solid phases and antigen sources (bovine/human), which probably also present non-identical epitopes and a different architecture in the antigen conjugation.

Furthermore, our results confirm the higher analytical sensitivity of CLIA platforms, not always corresponding to ELISA positivity. This discordance, in addition to being due to the different diagnostic sensitivities, may be related to the chemiluminescence signal itself, as well as the ability of the magnetic beads to bind antibodies with lower affinity, and could be solved by increasing CLIA *cut-off* values. The use of higher *cut-off* thresholds (e.g., >40 U/mL or >50 U/mL) for antiphospholipid antibody testing is strongly supported by clinicians; it improves the clinical significance and specificity of APS diagnosis. In fact, in our samples, at a *cut-off* > 50 U/mL, all groups showed a higher concordance. Similar results were reported in a comparative study by Lai et al., where ELISA/CLIA concordance was consistently high in subgroups with negative CLIA results, but more variable in CLIA-positive groups, particularly for IgG isotypes, and it increased when higher CLIA *cut-offs* were applied [20]. Moreover, the ISTH highlighted significant inter-platform differences in the aPLs measurement, recommending assay-specific considerations for moderate and high antibody levels to improve harmonization across analytical systems [19]. Together, these comparisons reinforce the notion that analytical discrepancies between CLIA and ELISA are not unique to our cohort but reflect broader challenges in aPLs testing, particularly regarding threshold selection and the interpretation of borderline results. Another strategy to exclude false positives could be to perform a confirmatory ELISA test. However, it should be noted that evaluating the distribution of the external quality verification (VEQs) highlights that many laboratories use ELISA tests, but there are several discrepancies within the same technology, confirming the need for better standardization.

Finally, our data, in accordance with the guidelines, show that ELISA appears to be more reliable compared to CLIA, particularly in CLIA-positive aCL and aβ2-GPI IgG detection. In this regard, our results provide further evidence that the LAC assay remains the most robust and accurate laboratory test for APS classification, finding a higher association between LAC results and aPLs IgG class ELISA detection than ELISA/CLIA.

Our study has some limitations, particularly the lack of diagnosis and the number of samples analyzed. Nevertheless, we would like to underline that this article is a comparison between methods; therefore, we believe that clinical information may not be necessary. Moreover, our aim was to categorize each group with the same numerousness, and each positive group was carefully selected on the basis of a single positivity, preventing possible interference with other auto-antibodies. In some cases, the frequency of positivity or negativity was low (i.e., negative LAC with positive aβ2-GPI IgM; positive LAC with negative aβ2-GPI IgG); however, the relatively low number of samples allowed for their detection on the same ELISA plate, avoiding the inter-assay variability.

In conclusion, we confirm that ELISA seems to be more reliable than CLIA for aPLs determination, but despite the fact that CLIA performances partly differ from ELISA, CLIA remains a valid clinical diagnostic alternative. Indeed, although ELISA is the reference method for classification criteria (which are used to define homogeneous groups of patients in research and clinical trials cohorts, aiming for high specificity to reduce false positives), this does not preclude the use of CLIA in the diagnosis of APS (which is instead used to confirm a suspected disease in an individual patient to guide treatment decisions, aiming for high sensitivity to detect any case of the disease).

Further studies are needed to shed light on the different parameters to be taken into account (such as variations in the solid phase shape: magnetic microparticles or microspheres) in order to harmonize data, to refine *cut-off* values, and to reach a greater standardization in the diagnostic laboratory methods.

## Figures and Tables

**Table 1:** Number of samples analyzed, mean age and number of males and females in the anti-cardiolipin and anti-β2 glicoprotein I IgG/IgM groups, divided into LAC-negative and LAC-positive.

	**LAC-NEGATIVE**				**LAC-POSITIVE**			
**aCL**	**IgG+**	**IgG−**	**IgM+**	**IgM−**	**IgG+**	**IgG−**	**IgM+**	**IgM−**
**Samples (*n*)**	22	22	24	22	22	22	25	24
**Age mean** **(SD)**	37.7 (±9.7)	46.6 (±14.9)	50.8 (±14.8)	45.6 (±12.9)	48.2 (±19.3)	63.0 (±11.6)	57.5 (±13.8)	55.8 (±17.0)
**Male** **Tot. number: 52**	3	6	6	6	8	7	6	10
**Female** **Tot. number: 131**	19	16	18	16	14	15	19	14
	**LAC-NEGATIVE**				**LAC-POSITIVE**			
**aβ2-GPI**	**IgG+**	**IgG−**	**IgM+**	**IgM−**	**IgG+**	**IgG−**	**IgM+**	**IgM−**
**Samples (n)**	21	22	17	22	23	19	21	21
**Age mean** **(SD)**	47.7 (±17.4)	43.1 (±12.8)	51.5 (±15.5)	45.5 (±13.8)	48.8 (±16.4)	59.7 (±12.8)	53.7 (±16.6)	55.0 (±15.7)
**Male** **Tot. number: 50**	8	6	5	6	8	7	4	6
**Female** **Tot. number: 116**	13	16	12	16	15	12	17	15

**Table 2:** Matrix template for the calculation of the concordance values in each group.

	CLIA+	CLIA−
**ELISA+**	a	b
**ELISA−**	c	d

**Table 3:** CLIA/ELISA concordance in the positive groups, comparing two different CLIA *cut-offs* with ELISA *cut-off* > 20 U/mL.

	LAC-NEGATIVE		LAC-POSITIVE	
	CLIA *Cut-Off* > 20 U/mL	CLIA *Cut-Off* > 50 U/mL	CLIA *Cut-Off* > 20 U/mL	CLIA *Cut-Off* > 50 U/mL
aCL IgG-positive (%)	4.5	14	50	65
aCL IgM-positive (%)	71	83	76	89
aβ2-GPI IgG-positive (%)	4.8	15	44	59
aβ2-GPI IgM-positive (%)	82	89	100	100

## Data Availability

The original contributions presented in this study are included in the article. Further inquiries can be directed to the corresponding author.

## Supplementary Materials

The following supporting information can be downloaded at https://www.mdpi.com/article/10.3390/diagnostics16111620/s1: Table S1: Main analytical and diagnostic characteristics of ELISA and CLIA tests, including antigen source, diagnostic sensitivity and specificity, coefficient of variation inter- and intra-assay and range of detection, as reported by the manufacturer; Table S2: Spearman correlation coefficient (ρ-rho) of the study groups.

## References

[1] Barreno-rocha S.G., Guzmán-silahua S., Rodríguez-dávila S.D.C., Gavilanez-chávez G.E., Cardona-muñoz E.G., Riebeling-navarro C., Rubio-jurado B., Nava-zavala A.H. (2022). Antiphospholipid Antibodies and Lipids in Hematological Malig-nancies. Int. J. Mol. Sci..

[2] Sammaritano L.R. (2020). Antiphospholipid syndrome. Best Pract. Res. Clin. Rheumatol..

[3] Sciascia S., Radin M., Bazzan M., Montaruli B., Cosseddu D., Norbiato C., Bertero M.T., Carignola R., Bacco B., Cassarino S.G. (2021). Antiphospholipid Antibodies and Infection: Non Nova Sed Nove. Front. Immunol..

[4] Gaspar P., Sciascia S., Tektonidou M.G. (2024). Epidemiology of antiphospholipid syndrome: Macro- and microvascular manifes-tations. Rheumatology.

[5] Dabit J.Y., Valenzuela-Almada M.O., Vallejo-Ramos S., Duarte-García A. (2022). Epidemiology of Antiphospholipid Syndrome in the General Population. Curr. Rheumatol. Rep..

[6] Hwang J.J., Shin S.H., Kim Y.J., Oh Y.M., Lee S.D., Kim Y.H., Choi C.W., Lee J.S. (2020). Epidemiology of antiphospholipid syndrome in korea: A nationwide population-based study. J. Korean Med. Sci..

[7] Wilson W.A., Gharavi A.E., Koike T., Lockshin M.D., Branch D.W., Piette J.C., Brey R., Derksen R., Harris E.N., Hughes G.R.V. (1999). International consensus statement on preliminary classification criteria for definite antiphospholipid syndrome: Report of an International Workshop. Arthritis Rheum..

[8] Miyakis S., Lockshin M.D., Atsumi T., Branch D.W., Brey R.L., Cervera R., Derksen R.H.W.M., Groot P.G.D.E., Koike T., Meroni P.L. (2006). International consensus statement on an update of the classification criteria for definite antiphospholipid syndrome (APS). J. Thromb. Haemost..

[9] Devreese K.M.J., Ortel T.L., Pengo V., de Laat B. (2018). Laboratory criteria for antiphospholipid syndrome: Communication from the SSC of the ISTH. J. Thromb. Haemost..

[10] Barbhaiya M., Zuily S., Naden R., Hendry A., Manneville F., Amigo M.C., Amoura Z., Andrade D., Andreoli L., Artim-Esen B. (2023). 2023 ACR/EULAR antiphospholipid syndrome classification criteria. Ann. Rheum. Dis..

[11] Favaloro E.J., Pasalic L., Selby R. (2024). Testing for the lupus anticoagulant: The good, the bad, and the ugly. Res. Pract. Thromb. Haemost..

[12] Devreese K.M.J., de Groot P.G., de Laat B., Erkan D., Favaloro E.J., Mackie I., Martinuzzo M., Ortel T.L., Pengo V., Rand J.H. (2020). Guidance from the Scientific and Standardization Committee for lupus anticoagu-lant/antiphospholipid antibodies of the International Society on Thrombosis and Haemostasis: Update of the guidelines for lupus anticoagulant detection and interpretation. J. Thromb. Haemost..

[13] Pizzuto M., Pelegrin P. (2020). Cardiolipin in Immune Signaling and Cell Death. Trends Cell Biol..

[14] Zeng H., Cai M., Xue H., Xie W., Long X. (2022). Prevalence and coagulation correlation of anticardiolipin antibodies in patients with COVID-19. Medicine.

[15] Hisada R., Atsumi T. (2023). An Antiphospholipid Antibody Profile as a Biomarker for Thrombophilia in Systemic Lupus Ery-thematosus. Biomolecules.

[16] Devreese K.M.J., Bertolaccini M.L., Branch D.W., de Laat B., Erkan D., Favaloro E.J., Pengo V., Ortel T.L., Wahl D., Cohen H. (2025). An update on laboratory detection and interpretation of antiphospholipid antibodies for diagnosis of antiphospholipid syndrome: Guidance from the ISTH-SSC Subcommittee on Lupus Anticoagulant/Antiphospholipid Antibodies. J. Thromb. Haemost..

[17] McDonnell T., Wincup C., Buchholz I., Pericleous C., Giles I., Ripoll V., Cohen H., Delcea M., Rahman A. (2020). The role of be-ta-2-glycoprotein I in health and disease associating structure with function: More than just APS. Blood Rev..

[18] Kumar S., Pozzi N. (2025). Understanding the structure of β2-glycoprotein I: New insights and future paths for antiphospholipid syndrome, Blood Vessels. Thromb. Hemost..

[19] Vandevelde A., Chayoua W., de Laat B., Gris J.C., Moore G.W., Musiał J., Zuily S., Wahl D., Devreese K.M.J. (2022). Semiquanti-tative interpretation of anticardiolipin and antiβ2glycoprotein I antibodies measured with various analytical platforms: Communication from the ISTH SSC Subcommittee on Lupus Anticoagulant/Antiphospholipid Antibodies. J. Thromb. Haemost..

[20] Lai E.E.N., Lim C.X.Q., Lau J.P.J., Chee Y.L., Chan S.S.W., Teo W.Z.Y., Yap E.S., Lee S.Y. (2025). Diagnosis of Antiphospholipid Syndrome by Chemiluminescent or Enzyme-Linked Immunosorbent Assay—A Comparison Study and Comprehensive Literature Review. Clin. Appl. Thromb..

[21] Wan L.Y., Gu J.Y., Liu T.T., Hu Q.Y., Jia J.C., Teng J.L., Sun Y., Liu H.L., Cheng X.B., Ye J.N. (2020). Clinical performance of automated chemiluminescent methods for anticardiolipin and anti-β2-glycoprotein I antibodies detection in a large cohort of Chinese patients with antiphospholipid syndrome. Int. J. Lab. Hematol..

[22] Hu C., Li S., Xie Z., You H., Jiang H., Shi Y., Qi W., Zhao J., Wang Q., Tian X. (2021). Comparison of Different Test Systems for the Detection of Antiphospholipid Antibodies in a Chinese Cohort. Front. Immunol..

[23] Ruffatti A., Olivieri S., Tonello M., Bortolati M., Bison E., Salvan E., Facchinetti M., Pengo V. (2008). Influence of different IgG an-ticardiolipin antibody cut-off values on antiphospholipid syndrome classification. J. Thromb. Haemost..

[24] Cross C., Cappola J. (2022). Anticardiolipin Antibodies Presenting With Acute Renal Infarction in a Healthy 26-Year-Old Female. Cureus.

[25] Nipu M.A.I., Kundu S., Alam S.S., Dina A.N., Hasan M.A., Khan M., Khalil M.I., Hossan T., Islam M.A. (2023). Anticardiolipin Antibodies in Patients with Cancer: A Case–Control Study. Cancers.

[26] Law E.C.Y., Leung D.T.M., Tam F.C.H., Cheung K.K.T., Cheng N.H.Y., Lim P.L. (2019). IgM Antibodies Can Access Cryptic An-tigens Denied to IgG: Hypothesis on Novel Binding Mechanism. Front. Immunol..

