# Supplementary material for: Comparison Between Chemiluminescent Assay and Enzyme-Linked ImmunoSorbent Assay Techniques for the Detection of Anti-Cardiolipin and Anti-β2 Glycoprotein I Antibody Values
Source: Diagnostics (Basel). 2026 May 25;16(11):1620. doi: 10.3390/diagnostics16111620 (PMC13256733; doi:10.3390/diagnostics16111620)
Supplement: Supplementary file 1 [file diagnostics-16-01620-s001.zip › diagnostics-4205753-supplementary.pdf]

Supplementary Table S1. Main analytical and diagnostic characteristics of ELISA and CLIA tests, including antigen source, diagnostic sensitivity and specificity, coefficient of variation inter- and intra-assay and range of detection, as reported by the manufacturer.

		aCL IgG	aCL IgM	aβ2-GPI IgG	aβ2-GPI IgM
**Antigen source**	ELISA	Purified bovine cardiolipin as an antigen and bovine β2-GPI protein as cofactor.	Purified bovine cardiolipin as an antigen and bovine β2-GPI protein as cofactor.	Purified β2-GPI protein from human serum	Purified β2-GPI protein from human serum
	CLIA	Purified bovine cardiolipin as an antigen and human β2-GPI protein as cofactor.	Purified bovine cardiolipin as an antigen and human β2-GPI protein as cofactor.	Purified β2-GPI protein from human serum	Purified β2-GPI protein from human serum
**Sensitivity (%)**	ELISA	96.6	94.0	20.8	83.3
	CLIA	54.3	33.7	64.1	29.3
**Specificity (%)**	ELISA	98.7	97.8	100	93.6
	CLIA	95.6	94.8	90.8	95.2
**Coefficient of variation (CV) inter-assay (%)**	ELISA	5.5	12.0	4.0	3.0
	CLIA	6.2	3.2	6.2	4.7
**Coefficient of variation (CV) intra-assay (%)**	ELISA	4.0	12.2	3.3	2.7
	CLIA	4.6	1.8	4.6	3.3
**Range of detection (U/mL)**	ELISA	0-150.0	0-150.0	0-150.0	0-150.0
	CLIA	2.6-2024.0	1.0-774.0	6.4-6100.0	1.1-841.0

Supplementary Table S2. Spearman correlation coefficient (ϱ-rho) of the study groups.

	LAC-NEGATIVE	LAC-POSITIVE
**aCL IgG positive**	rho = 0.155<br>95% CI (-0.285-0.541)<br>p = 0.4901	rho = 0.766<br>95% CI (0.524-0.893)<br>p < 0.0001
**aCL IgG negative**	rho = -0.027<br>95% CI (-0.444-0.399)<br>p = 0.902	rho = 0.0401<br>95% CI (-0.388-0.454)<br>p = 0.859
**aCL IgM positive**	rho = 0.423<br>95% CI (0.0239-0.706)<br>p = 0.0393	rho = 0.842<br>95% CI (0.669-0.928)<br>p < 0.0001
**aCL IgM negative**	rho = 0.492<br>95% CI (0.088-0.756)<br>p = 0.0201	rho = 0.648<br>95% CI (0.331-0.834)<br>p = 0.0006
**aβ2-GPI IgG positive**	rho = 0.221<br>95% CI (-0.233-0.596)<br>p = 0.3352	rho = 0.793<br>95% CI (0.567-0.909)<br>p < 0.0001
**aβ2-GPI IgG negative**	rho = 0.423<br>95% CI (0.001-0.717)<br>p = 0.0498	rho = 0.121<br>95% CI (-0.353-0.545)<br>p = 0.6224
**aβ2-GPI IgM positive**	rho = 0.072<br>95% CI (-0.423-0.534)<br>p = 0.7826	rho = 0.803<br>95% CI (0.568-0.917)<br>p < 0.0001
**aβ2-GPI IgM negative**	rho = 0.531<br>95% CI (0.140-0.778)<br>p = 0.0111	rho = 0.792<br>95% CI (0.547-0.912)<br>p < 0.0001
